# Rapid greening response of China’s 2020 spring vegetation to COVID-19 restrictions: Implications for climate change

**DOI:** 10.1126/sciadv.abe8044

**Published:** 2021-08-25

**Authors:** Fenzhen Su, Dongjie Fu, Fengqin Yan, Han Xiao, Tingting Pan, Yang Xiao, Lu Kang, Chenghu Zhou, Michael Meadows, Vincent Lyne, John P. Wilson, Na Zhao, Xiaomei Yang, Gaohuan Liu

**Affiliations:** 1 State Key Laboratory of Resources and Environmental Information System, Institute of Geographic Sciences and Natural Resources Research, Chinese Academy of Sciences, Beijing 100101, China.; 2 College of Resources and Environment, University of Chinese Academy of Sciences, Beijing 100049, China.; 3 School of Geography and Ocean Science, Nanjing University, Nanjing 210023, China.; 4 Collaborative Innovation Center of South China Sea Studies, Nanjing University, Nanjing 210093, China.; 5 Department of Environmental and Geographical Science, University of Cape Town, Rondebosch 7701, South Africa.; 6 School of Geographic Sciences, East China Normal University, Shanghai 200241, China.; 7 College of Geography and Environmental Sciences, Zhejiang Normal University, Jinhua 321004, China.; 8 IMAS-Hobart, University of Tasmania, Hobart, Tasmania 7004, Australia.; 9 Spatial Sciences Institute, University of Southern California, Los Angeles, CA 90089, USA.

## Abstract

The 2019 novel coronavirus pandemic (COVID-19) negatively affected global public health and socioeconomic development. Lockdowns and travel restrictions to contain COVID-19 resulted in reduced human activity and decreased anthropogenic emissions. However, the secondary effects of these restrictions on the biophysical environment are uncertain. Using remotely sensed big data, we investigated how lockdowns and traffic restrictions affected China’s spring vegetation in 2020. Our analyses show that travel decreased by 58% in the first 18 days following implementation of the restrictions across China. Subsequently, atmospheric optical clarity increased and radiation levels on the vegetation canopy were augmented. Furthermore, the spring of 2020 arrived 8.4 days earlier and vegetation 17.45% greener compared to 2015–2019. Reduced human activity resulting from COVID-19 restrictions contributed to a brighter, earlier, and greener 2020 spring season in China. This study shows that short-term changes in human activity can have a relatively rapid ecological impact at the regional scale.

## Teaser

Decreased human activities from COVID-19 containment measures caused a brighter, earlier, and greener 2020 spring in China.

## INTRODUCTION

The outbreak of the coronavirus disease 2019 (COVID-19) negatively affected people’s health and the economic and social development of countries worldwide (*1*–*5*). To contain COVID-19, governments globally implemented intensive restrictions, including lockdowns, constraints on transport, social distancing, and limitations on movement (*6*–*10*). The rapid and comprehensive restrictions assisted in containing COVID-19 (*10*) and created an unprecedented regional-scale natural experiment on the environmental impacts of changes in human activity. These impacts include reduced anthropogenic emissions and, in turn, decreased aerosol optical depth (AOD), both of which are known to influence radiation and, therefore, vegetation growth (*11*–*15*). Several studies described the effects of COVID-19 controls on anthropogenic emissions and atmospheric quality (*16*–*19*). Thus far, however, global and regional ancillary effects on vegetation have not been investigated quantitatively. This study therefore aims to assess, at a regional scale, how measures taken to contain COVID-19 influenced atmospheric conditions and a range of physical and biological attributes in the spring of 2020 across China. The implications of these findings for climate change research are considered with a view to better informing researchers and policy-makers of future possible policies, actions, and regional outcomes.

Remotely sensed (RS) big data and population flow data were used to track the effect of COVID-19 restrictions on the spatiotemporal patterns of changes in human activity, atmosphere, radiation, and vegetation through the first 4 months of 2020 in China. Baidu location-based services data were used to calculate an index of population movement intensity [migration scale index (MSI)], which can be considered as a proxy for human activity in general. RS big data were used next to determine changes in the atmosphere reflected in nitrogen dioxide (NO_2_), AOD, and photosynthetically active radiation (PAR) and then changes in vegetation as indicated by leaf area index (LAI) and gross primary productivity (GPP). We then compared the various indices for the spring months of recent years to understand the effects of imposed restrictions on anthropogenic emissions, atmospheric conditions, and vegetation growth.

## RESULTS

### The effect of prevention measures on population flow intensity

**Fig. 1:**
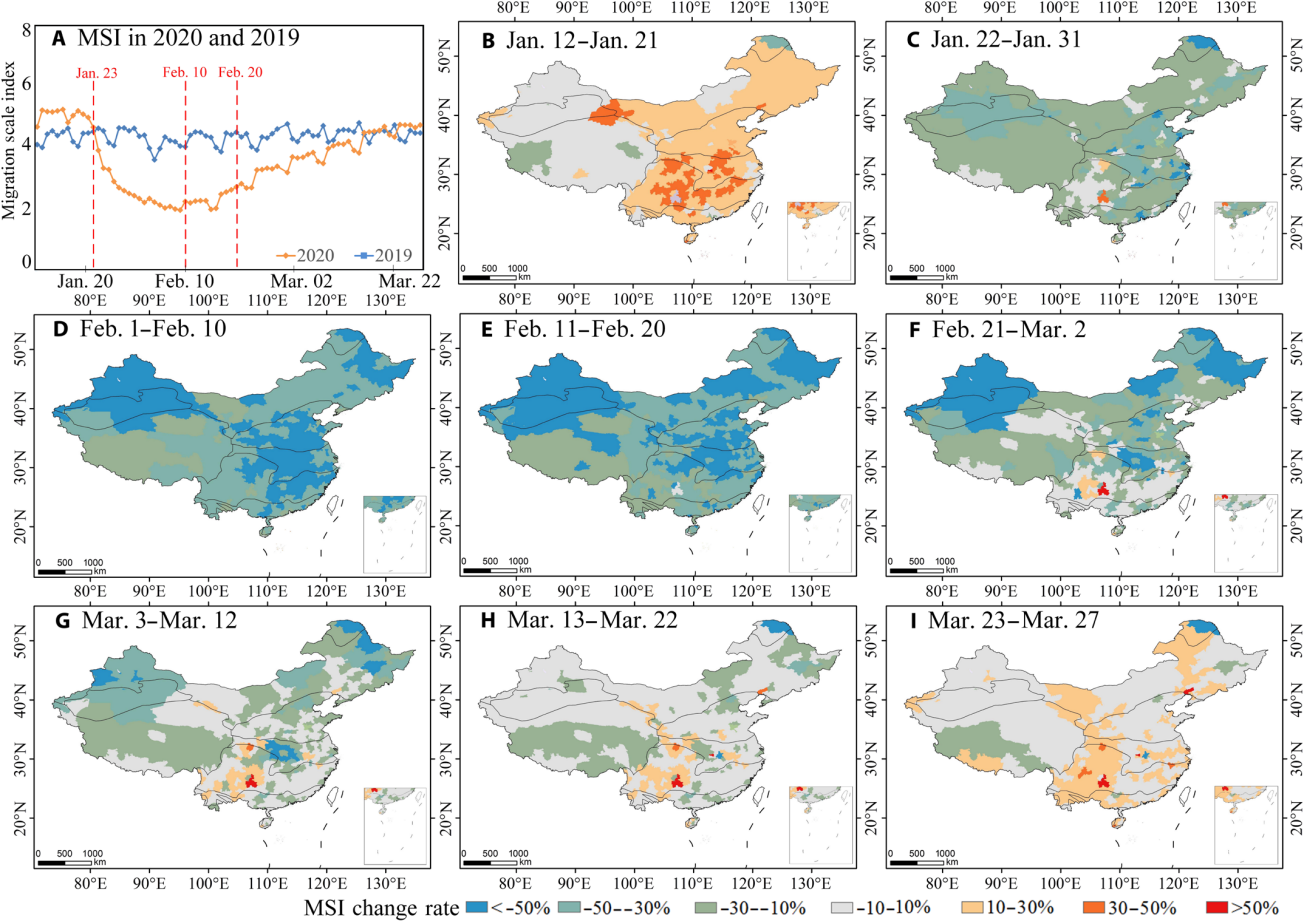
Comparison of MSI for the same periods between 2019 and 2020. (**A**) National temporal trend of MSI from January to March; (**B** to **I**) spatial pattern of MSI change rates at the prefecture level for different time periods between 2019 and 2020 (blue and green represent decreased MSI, while orange and red represent increased MSI). Boundaries are the climate zones of China, defined in fig. S1.

In responding to the confirmed outbreak of COVID-19, China initiated first-level public health responses across 31 provinces and municipalities from 23 January 2020 (*20*, *21*). As part of the lockdowns, authorities implemented strict controls on the aggregation of people and restrictions on transport between adjacent districts or prefectures. On February 10, the government further strengthened epidemic prevention and control through stricter limits on movement in communities (*22*). Changes in MSI show that COVID-19 restrictions had a rapid and substantive impact on population flow intensity. Between 23 January and 9 February 2020, the overall national MSI decreased by 58% (see Fig. 1A). During the period of February 10 to 24, the MSI trended upward slowly, although it remained at much lower levels than in the equivalent period of previous year and only approached 2019 values around March 16 (Fig. 1A). Spatially, 10-day mean values of MSI (Fig. 1, B to I) reflected a widespread downward trend, except for prefectures in the sparsely populated plateau climate zone (climate zones are defined in fig. S1), which was the last zone to initiate first-level public health responses, on 29 January 2020 (*21*).

### Changes in atmospheric emissions

NO_2_ and AOD were used to investigate the impact of the COVID-19 control measures on atmospheric quality. NO_2_ is primarily derived from fossil fuel consumption and is one of the main atmospheric pollutants involved in the formation of photochemical smog (*23*); AOD measures the aerosol components such as smog, atmospheric particles, and dust. AOD is a direct indicator of the degree to which aerosols impede optically effective radiation (*24*). The decline in nationwide human activity shown in Fig. 1 was accompanied by marked regional reductions in NO_2_ (fig. S2) and AOD (fig. S3), most prominently in areas with large resident populations (e.g., the southern temperate and northern subtropical zones).

Atmospheric NO_2_ concentrations in 2020 exhibited an obvious downward trend from January 1 to April 9 (fig. S2), with the largest decrease of 35.35% in February compared to 2019 (Table 1). For 10-day NO_2_ in 2020, tropospheric NO_2_ vertical columns (TvcNO_2_) showed an obvious reduction during the period January 21 to 30 and gradually increased from February 20 to March 20 (fig. S2). Nationally, lowest values of TvcNO_2_ occurred during the three 10-day periods (21 January to 19 February 2020) coinciding with the sharpest decline in national human activity (see MSI in Fig. 1). Consistent with other studies (*13*, *23*, *25*, *26*), the most notable reduction (fig. S2) occurred in highly populated and developed parts of the country.

**Table 1:** Parameters for human and environmental changes between 2020 and the previous 5 years (2015–2019) for China’s 2020 spring.

**Parameter**	**February**			**March**			**April**		
	**2015–2019**	**2020**	**Change (%)**	**2015–2019**	**2020**	**Change (%)**	**2015–2019**	**2020**	**Change (%)**
MSI*	4.26	2.48	−41.68	4.47	4.16	−6.88			
NO_2_*	90.37	58.42	−35.35	88.41	72.85	−17.60	83.25	79.58	−4.40
AOD	520.72	500.68	−3.85	583.37	571.04	−2.11	511.73	504.26	−1.46
PAR^†^	181.09	187.43	3.50	243.97	244.69	0.30	292.00	296.62	1.58
LAI	0.59	0.62	4.98	0.63	0.65	2.97	0.90	0.99	10.11
GPP	8.14	8.91	9.36	11.73	11.39	−2.90	16.84	18.80	11.63

*Monthly differences in MSI and NO_2_ are the differences between the values in 2020 and 2019.

†The monthly differences in PAR are the differences between the values in 2020 and 2018–2019.

AOD increased initially by 7.87% in January 2020 relative to 2015–2019, and lower AOD values were observed mainly in remote mountainous regions such as Guizhou province (fig. S3). However, once control measures were applied from late January, AOD values declined across China (February). Consistent with another study (*18*), the spatial pattern of AOD change was similar to NO_2_, with especially noteworthy declines in the densely populated areas of the southern temperate zone in March and then across the northern subtropical, mid-subtropical, and southern subtropical zones in April (fig. S3).

### Changes in radiation

**Fig. 2:**
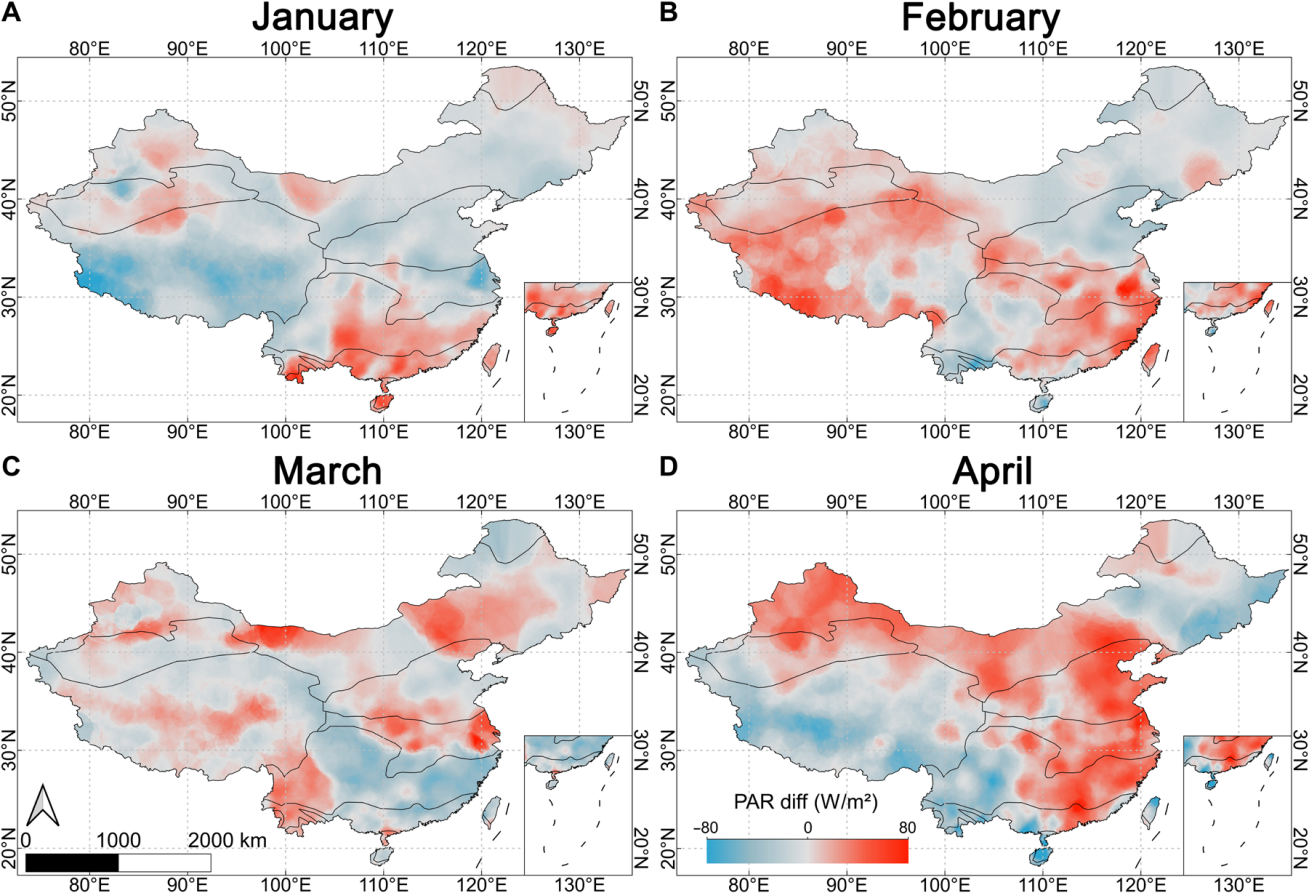
Spatial distribution of mean monthly PAR differences between year 2020 and years 2018 and 2019 (unit: W/m^2^; blue represents decreased PAR, while red represents increased PAR). (**A** to **D**) PAR differences from January to April.

PAR is radiation with wavelengths between 400 and 700 nm, which is in the range that vegetation harnesses via the process of photosynthesis (*27*). Spatially, PAR differences were small in most areas in January while higher PAR values were observed in February 2020, especially in southern China and the plateau climate zone. Elevated PAR values then occurred in northern China in March and April (Fig. 2). Compared to 2018–2019, monthly PAR differences began to increase from February, indicating that the spring months of 2020 were brighter than previous years. The largest relative change was observed between January and February, corresponding to the peak in lockdown restrictions.

### The response of vegetation

LAI, defined as the one-sided leaf area coverage of vegetation per unit ground surface area (*28*), was used here to investigate phenology and greenness of vegetation growth during spring. GPP is the amount of carbon fixed during photosynthesis (*29*, *30*) and was used as an auxiliary proxy to represent spring vegetation growth in general.

Spatially, LAI values in January 2020 were lower than 2015–2019 in most areas (fig. S4). However, higher LAI values were observed for 2020 from February to April compared to the previous 5 years (fig. S4), especially in April. Although there are variations between months, more positive GPP values were observed from January to April in 2020 compared to 2015–2019 (fig. S5), eventually exhibiting nationwide higher GPP values in April except for the plateau climate zone.

**Fig. 3:**
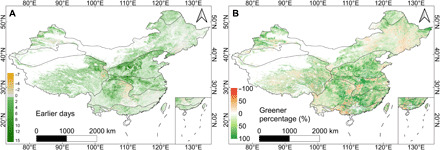
Spatial distribution of earlier and greener spring in 2020 across China. (**A**) Earlier days. Green/red represents number of days earlier/later than the previous 5 years (2015–2019). (**B**) Greener percentage (%). Green/red represents the percentage increase/decrease of LAI at base date compared to the previous 5 years (2015–2019).

To investigate changes in phenology in the spring of 2020, we compared the LAI values at base dates for the start of spring with those from 2015–2019. Details relating to the estimation of the base dates are given in Materials and Methods. With 6 April 2020 estimated as the base date for China as a whole, the onset of spring in 2020 occurred 8.4 days earlier, especially in the northern subtropical zone (base date, April 2) where it occurred 9.1 days earlier (Fig. 3A and table S1).

The change in greenness was computed by comparing LAI values for 2020 with those of 2015–2019 at the equivalent base dates. Overall, LAI at the base date increased by 17.45% compared to 2015–2019, indicating a substantially greener spring season in 2020. Spatially, most areas were greener in the spring of 2020 than 2015–2019 (Fig. 3B). All seven climate zones were greener in the spring of 2020 detailed as follows (% increase, region): 32.71%, northern subtropical zone; 30.27%, southern temperate zone; 28.76%, mid-subtropical zone; 21.67%, southern subtropical zone; 20.04%, plateau climate zone; 14.83%, northern temperate zone; and 7.36%, mid-temperate zone (table S1).

To assess the significance of the changes in LAI, first, we calculated the difference in each pixel value between pairs of adjacent years from 2003–2019 to ascertain whether LAI differences are normally distributed. The results (table S2) show that LAI values in most areas (>80%) across the six climate zones are indeed normally distributed. Statistical analysis was then applied to identify the areas that exhibited substantial changes in 2020 relative to 2019. The results indicate that China, in general, was notably greener in 2020, especially in February and April. Of those pixels classified as greener in the spring of 2020 compared to previous years, more than 60% of these were notably greener, a proportion that exceeded 75% for February (fig. S6 and table S3). Vegetation growth increases were also recorded in GPP and, although there are variations between months, fig. S6, in general, indicates more positive spring values for 2020 compared to the previous years. Differences in monthly GPP values were +0.76, −0.34, and +1.96 g C/m^2^ per 8 days corresponding to February, March, and April, respectively (Table 1).

## DISCUSSION

Our integrated spatiotemporal analyses show that a brighter, earlier, and greener spring of 2020 in China occurred after intensive restrictions were imposed to constrain the outbreak of COVID-19. The spatial distribution of changes in human activity, atmospheric emission, radiation, and vegetation from January to April (Figs. 1 to 3 and figs. S2 to S5) was logically consistent and similar. Moreover, the temporal changes in these parameters are consistent and supported by the monthly statistical summaries of the spatial patterns (Table 1). Reduction in short-term human activity positively affected the spatiotemporal pattern of vegetation growth indirectly via a logical sequence of linkages as follows: Less human activity (decreased MSI) led to clearer skies (lower NO_2_ and AOD), allowing more radiation (increased PAR) to reach the canopy that, in turn, enhanced vegetation growth (increased LAI and GPP).

Previous studies have reported declined NO_2_ and AOD in response to reduced human activities (*18*, *23*). We extended these studies and investigated causal links using Granger causality analysis, which indicated causal links between AOD and PAR and between PAR and LAI (table S4). The expected strong association between PAR and vegetation growth (*28*, *31*, *32*) is observed in higher values of LAI resulted from enhanced spring radiation. This is consistent with our correlation analysis showing that enhanced radiation notably correlated with spring phenology in 2020 (figs. S7 to S10) especially in the subtropical zones. We found that increased PAR raised LAI such that the spring of 2020 in China arrived earlier (8.4 days) and was greener (17.45%) than 2015–2019. Our results extend previous studies that have reported declining NO_2_ and AOD in response to reduced human activities as well as the effect of AOD on radiation (*18*, *23*–*25*). Overall, our extended spatiotemporal analyses, correlations with changes in climatic factors, and causality demonstrate that intensive restrictions to constrain the spread of COVID-19 enhanced radiation and caused a brighter, earlier, and greener 2020 spring in China.

Despite the obvious negative impacts of COVID-19 on human well-being, we traced substantial positive impacts on atmospheric optical clarity that subsequently enhanced vegetation growth. These results demonstrate that reductions in the intensity of human activity may induce rapid responses in the wider environment, including vegetation. Although numerous studies have reported the global climate response to anthropogenic emissions at decadal to centennial time scales, this study reveals that on temporal scales of weeks to months, reduction in human activities can have rapid positive effects on the environment across large spatial scales. Therefore, reducing human activity not only affects climate change over the long term but also may have a more short-term impact that, with appropriate public education, can help to raise awareness of the positive effects of pollution control. The unprecedented and unique natural experiment provided by the restrictions imposed to contain COVID-19 exposes the close connections between human activity and vegetation growth as well as advances modeling and analytical insights into the impacts of short-term anthropogenic changes.

## MATERIALS AND METHODS

### Calculation of different parameters

The Python programming and Google Earth Engine platform (*33*) was used in this study to calculate the following parameters.

#### Population flow intensity

Using Python programming, the daily MSI of each city in China was mined from the Baidu migration platform (http://qianxi.baidu.com/), a large data visualization project developed by Baidu to characterize the population flow intensity (*25*, *34*, *35*). Considering the lockdown time and the lag effect on environment changes, 12 January to 27 March 2020 was selected as the study period and compared against the same period in 2019. The national daily MSI was obtained by the daily average MSI of all prefecture-level cities. A 10-day difference in MSI (2020–2019) of all prefecture-level cities was calculated between 2020 and 2019 (Fig. 1).

#### Ten-day NO_2_

Atmospheric NO_2_ values were derived from the “total vertical column of nitrogen dioxide (TvcNO_2_)” based on the Sentinel-5 Precursor (*36*), defined by the ratio of the slant column density of NO_2_ and the total air-mass factor

$$N_{v}=N_{s}/M$$ (1)

where *N*_v_ is the total vertical column density, *M* is the total air-mass factor, and *N*_s_ is the total slant column density. The average value of TvcNO_2_ in each 10-day period from 1 January to 9 April 2020 was used to generate the 10 nationwide maps shown in fig. S2A. The national mean values of TvcNO_2_ for 2020 and 2019 are compared in the histograms of fig. S2B.

#### AOD difference

AOD was derived from the “Corrected aerosol optical depth (Land) at 0.47, 0.55, and 0.66 microns wavelength: Mean of Level-3 QA Weighted Mean” band (*37*) in the moderate-resolution imaging spectroradiometer (MODIS) MOD08_E3 product. Images were preprocessed to remove null values and using only those within the range (−100 to 5000), as recommended in the user guide (https://lpdaac.usgs.gov/documents/110/MCD19_User_Guide_V6.pdf). Monthly images were generated from the mean value of each pixel. Monthly differences between the average values in 2020 and the corresponding average values in the previous 5 years (2015–2019) were then compared (Table 1 and fig. S3).

#### Photosynthetically active radiation

Monthly PAR was calculated by averaging the daily values from the MODIS Terra-and-Aqua combined PAR gridded level 3 product (MCD18A2). Monthly differences in PAR between 2020 and the two prior years (2018 and 2019) were calculated, and data gaps were filled using kriging interpolation (Fig. 2).

#### Leaf area index

LAI is a dimensionless quantity that characterizes vegetation cover and is defined as the relative one-sided projected area of leaves over a unit area of land (m^2^ m^−2^) (*28*, *38*–*40*). This parameter is used to estimate photosynthetic primary production and evapotranspiration and as a reference tool for estimating vegetation growth (*41*–*43*).

MODIS Terra-and-Aqua combined fraction of PAR and LAI gridded level 4 product (MCD15A3H) were used in this study. The monthly composited LAI, via 4-day average values, was used to calculate monthly differences between 2020 and previous 5 years (2015–2019) (fig. S4).

#### Gross primary productivity

The MODIS GPP (MOD17A2H) data were used to calculate monthly differences between the GPP values in 2020 and the mean values in the five prior years (2015–2019) (fig. S5).

#### Spring base date

Harmonic time series filtering was performed on the 2015–2019 LAI data to obtain a smoothed LAI. The date when the smoothed LAI was at 50% of the average 5-year maximum value (LAI_base_) was chosen as a nominal reference point marking the base date. The mean value of the base dates of all the pixels in China (April 6) was set as the base date for the whole of China. To more accurately investigate earlier/later days of spring onset in each climate zone, the base date in each climate zone was also calculated and used to produce the map in Fig. 3A.

#### Earlier/later days

To eliminate LAI fluctuations and null values, the average value of 4 days before and after the base date was selected to compute the LAI value at the base date. The 2015–2019 average value of LAI was chosen as the comparison value and was subtracted from the base date LAI value of 2020 to calculate the date corresponding to the minimum difference. This allowed the earlier/later days to be calculated for each pixel in 2020. The earlier/later days for the whole of China, and for each climate zone, were obtained by calculating the average values in the corresponding area. The interquartile range (IQR), equaling the difference between the 25th and 75th percentiles, is typically used to characterize variability when the shape of the distribution is skewed or outliers are present (*44*). In our study, IQR of pixel values was used in calculating the earlier/later days.

#### Greenness percentage

The percentage changes of LAI on the base date were calculated to represent the greenness change in percent (PG) as follows

$$\text{PG}=({\text{LAI}}_{2020}-{\text{LAI}}_{2015-2019})/{\text{LAI}}_{2015-2019}\times 100\%$$ (2)

where PG is the greenness change in percent and LAI_2020_ is the LAI value at the base date in 2020. LAI_2015–2019_ is the LAI value of the corresponding day of year in the previous 5 years (2015–2019). To eliminate LAI fluctuations and null values, the average value of 4 days before and after the base date was selected as the LAI value of the base date in 2020. Results of the greenness differences are shown in Fig. 3B.

### Significance testing

The differences between LAI across adjacent years from 2003 to 2019 at the same pixels exhibited a normal distribution (table S2), allowing us to put confidence intervals on adjacent-year differences for each pixel. Adjacent-year 2019–2020 differences were compared with the confidence intervals for each pixel. The significance of differences was tested with reference to the upper/lower 95% limits of the confidence interval to identify the notably different pixels. Results of notably higher and nominally higher LAI regions of spring months were shown in fig. S6.

## SUPPLEMENTARY MATERIALS

Supplementary material for this article is available at http://advances.sciencemag.org/cgi/content/full/7/35/eabe8044/DC1
